# Supplementary material for: A Soybean Sucrose Non-Fermenting Protein Kinase 1 Gene, GmSNF1, Positively Regulates Plant Response to Salt and Salt–Alkali Stress in Transgenic Plants
Source: Int J Mol Sci. 2023 Aug 5;24(15):12482. doi: 10.3390/ijms241512482 (PMC10419833; doi:10.3390/ijms241512482)
Supplement: Supplementary file 1 [file ijms-24-12482-s001.zip › ijms-2523564-supplementary.pdf]

Table S1: Primers used in qRT PCR analysis

Gene	Sequence (5'-3')	Purpose
GmActin11	F: GCGGGAAATTGTAAGGGATGT	Internal reference gene for *GmSNF1* expression analysis
	R: TCGCCAATAGTGATGACCTG	in soybean hairy roots and gene silenced plants
GmSNF1	F:TGAGCTCCAACATTGGTGTGG	*GmSNF1* gene expression analysis
	R:ACACGCACAGGGCTAAGGATTC	
GmABI1	F:TGCCAGGGCTACTGATTTGG	*GmABI1* gene expression analysis
	R:CCCCATAGCGGGGTACAATC	
GmABI2	F:AAACGGTCGCAGGTTCAGAT	*GmABI2* gene expression analysis
	R:GCCACAGCAAGAGGATCAGT	
GmCAT	F:ATGAGATCCGCAGTGTCTGG	*GmCAT* gene expression analysis
	R:GGCCTCGTGCTGAGATGAGA	
GmSNF4	F:GTGGAAGCTAATCCGAGGA	*GmSNF4* gene expression analysis
	R:GACTGGGGAAGCAACTCATGC	
GmERF7	F:CTGCATTGGCGTCTTTGCTCAG	*GmERF7* gene expression analysis
	R:TCATAGGGTTTCCAGCAGCATCC	
GmPKS4	F:GTTCGATTTGTCGCCGTTGT	*GmPKS4* gene expression analysis
	R:CTTCCAATCTCGAAATCACGCT	

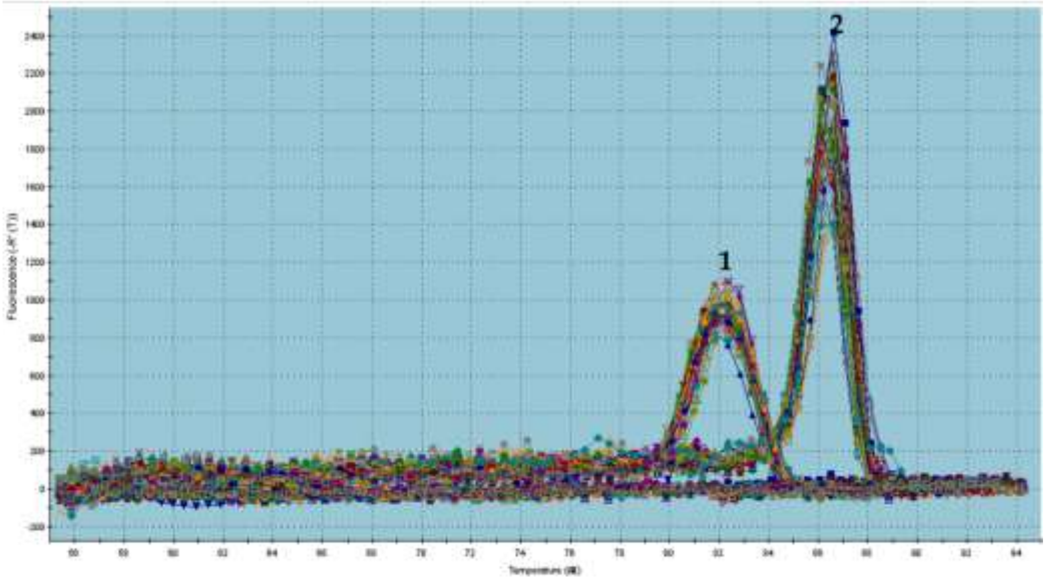

Figure S1 Melting curve analysis of *GmSNF1* gene in soybean roots under salt, salt-alkali, drought, and ABA stresses at 0 h, 1 h, 3 h, 6 h, 12 h, 24 h ,48 h. **1** represents for *GmActin11* gene; **2** represents for *GmSNF1* gene.

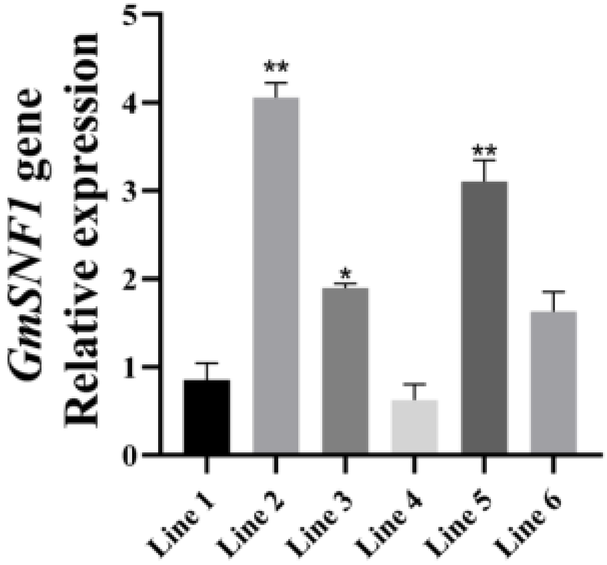

Figure S2 Checking for *GmSNF1* gene expression level in the six selected T3 homozygotes *Arabidopsis* lines. 25-day-old *Arabidopsis* samples were used. Data represent mean and standard deviation of three repeats ($n = 3$). Significant differences were determined by unpaired two-sided Student's t-tests (*, $P < 0.05$; **, $P < 0.01$).

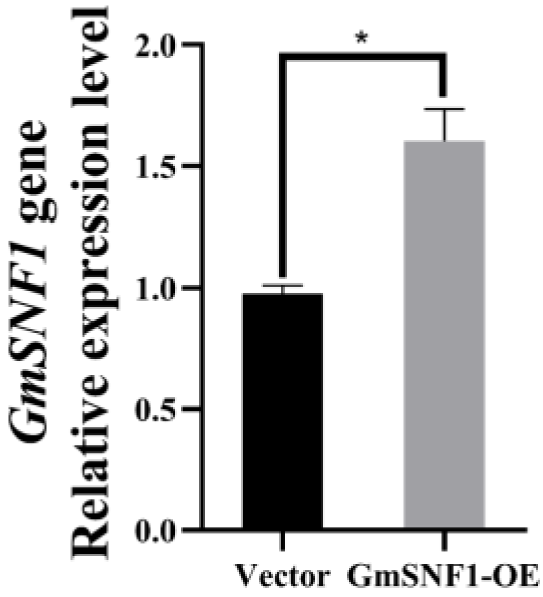

Figure S3 Expression of *GmSNF1* in vector controls and transgenic soybeans. Data represent mean and standard deviation of three repeats (n = 3). Significant differences were determined by unpaired two-sided Student's t-tests (*, $P < 0.05$).

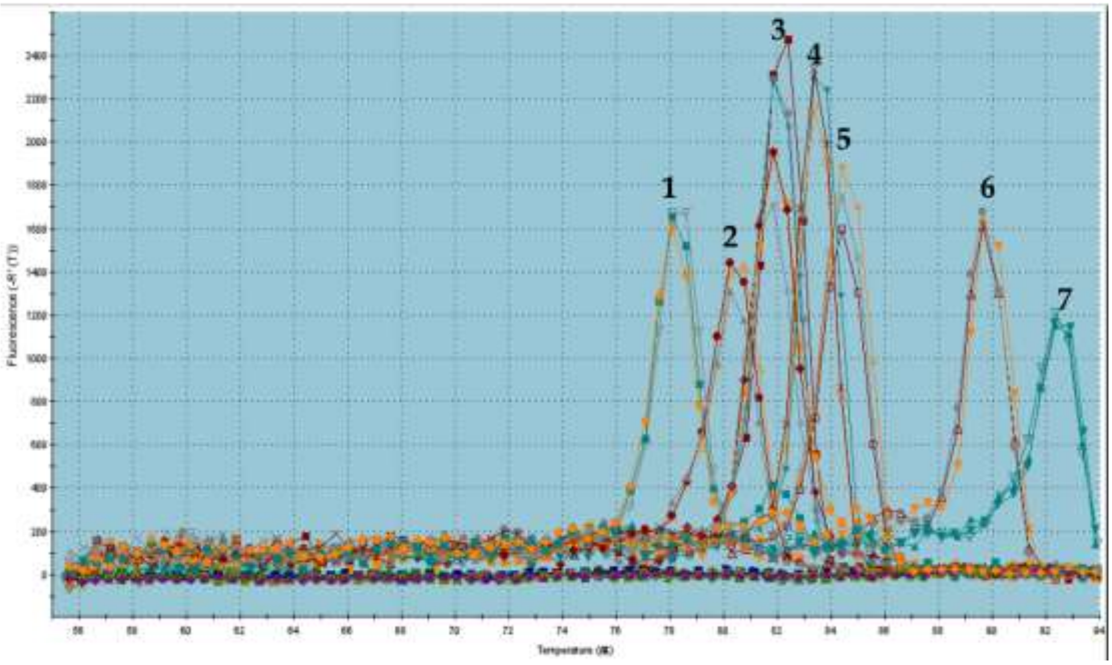

Figure S4 Melting curve analysis of some stress-related genes in GmSNF1-OE soybean plants under salt-alkali stress. **1** represents for *GmPKS4* gene; **2** represents for *GmActin11* gene; **3** represents for *GmABI1* gene; **4** represents for *GmABI2* gene; **5** represents for *GmSNF4* gene; **6** represents for *GmCAT* gene; and **7** represents for *GmERF7* gene.

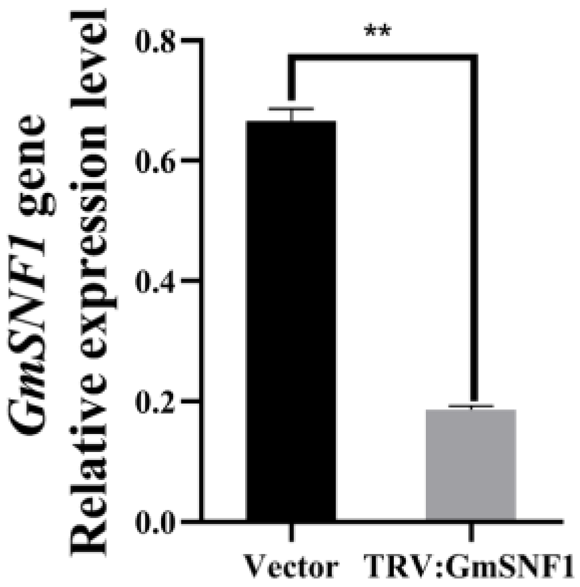

Figure S5 Expression of *GmSNF1* in vector controls and gene-silenced soybeans. Data represent mean and standard deviation of three repeats (n = 3). Significant differences were determined by unpaired two-sided Student's t-tests (**, P < 0.01).

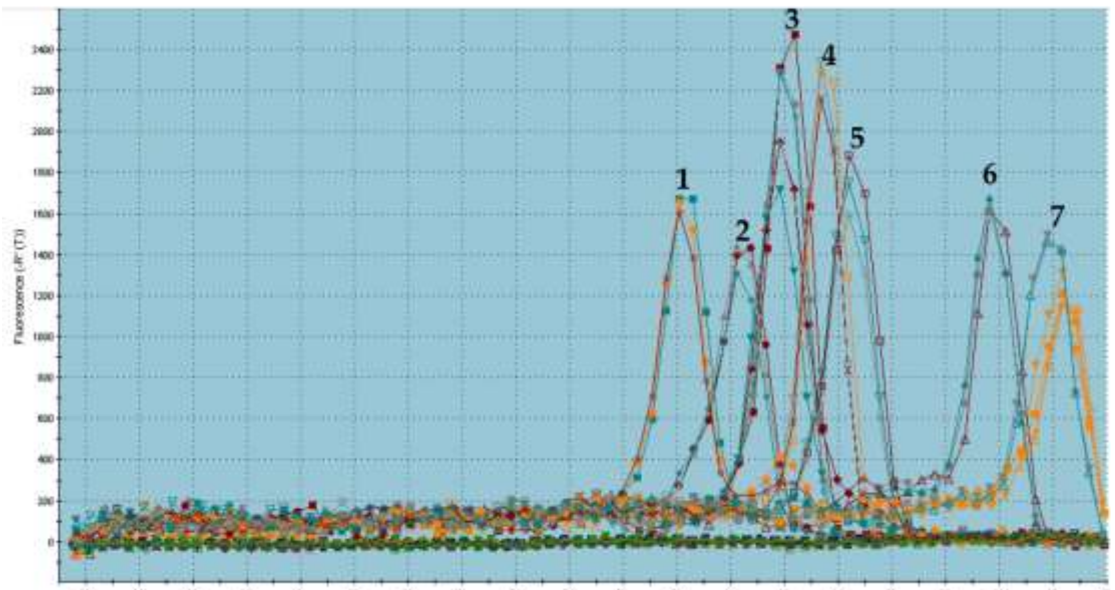

Figure S6 Melting curve analysis of some stress-related genes in *GmSNF1* gene-silenced soybean plants under salt-alkali stress. **1** represents for *GmPKS4* gene; **2** represents for *GmActin11* gene; **3** represents for *GmABI1* gene; **4** represents for *GmABI2* gene; **5** represents for *GmSNF4* gene; **6** represents for *GmCAT* gene; and **7** represents for *GmERF7* gene.

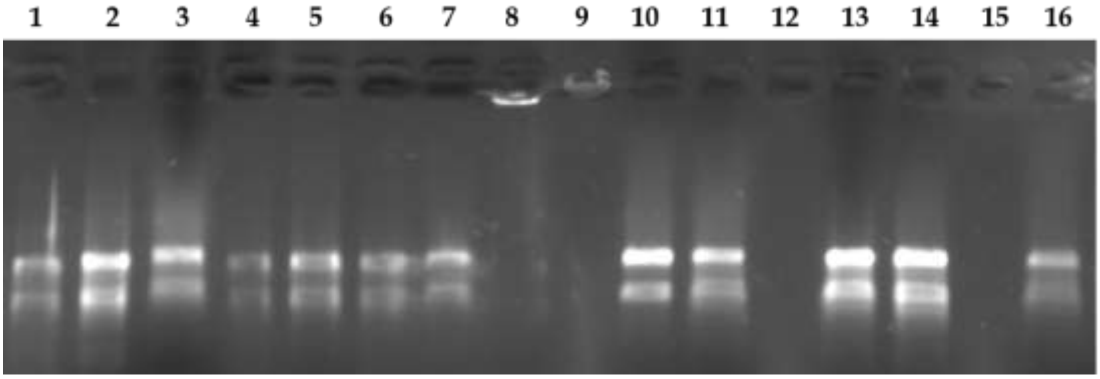

Figure S7 Extraction of RNA from soybean roots under salt and salt-alkali stress. 1, 2, 3, 4, 5, 6, 7 are salt treated at 0 h, 1 h, 3 h, 6 h, 12 h, 24 h, 48 h; 10, 11, 13, 14, 16 are salt-alkali treated at 1 h, 3 h, 6 h, 12 h, 24 h, respectively (The degraded RNA of some samples had been re-extracted.).

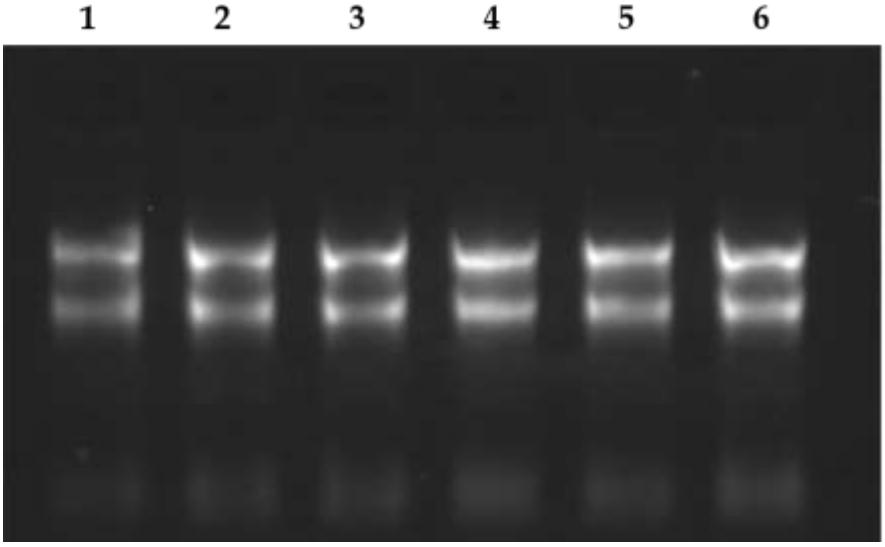

Figure S8 Extraction of RNA from soybean leaves under salt stress. 1, 2 ,3 ,4 ,5 ,6 represent for leaves treated under salt stress at 1 h, 3 h, 6 h, 12 h, 24 h ,48 h, respectively.

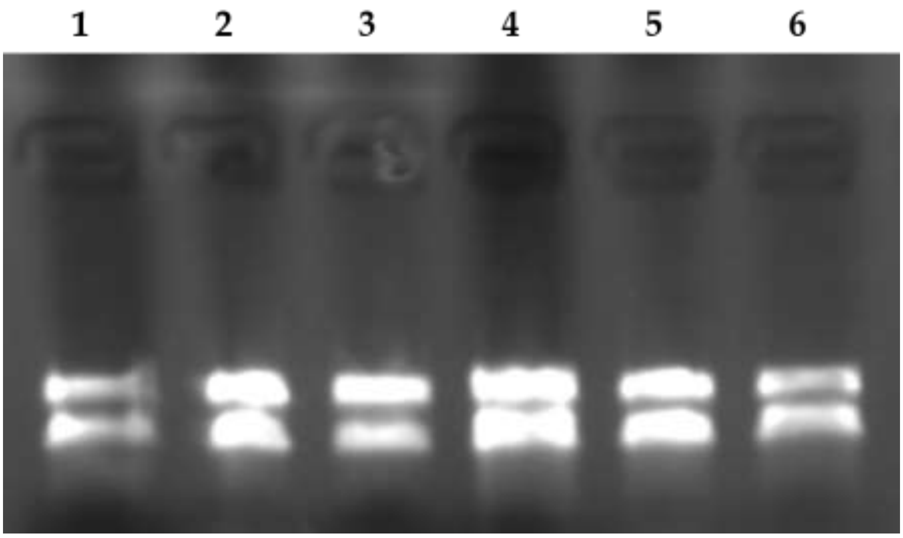

Figure S9 Extraction of RNA from GmSNF1-OE soybean plants under salt and salt-alkali stresses. 1, 3, and 5 represent for control plants under no treatment, salt treatment, and salt-alkali treatment, respectively; 2, 4, and 6 represent for GmSNF1-OE plants under no treatment, salt treatment, and salt-alkali treatment, respectively.

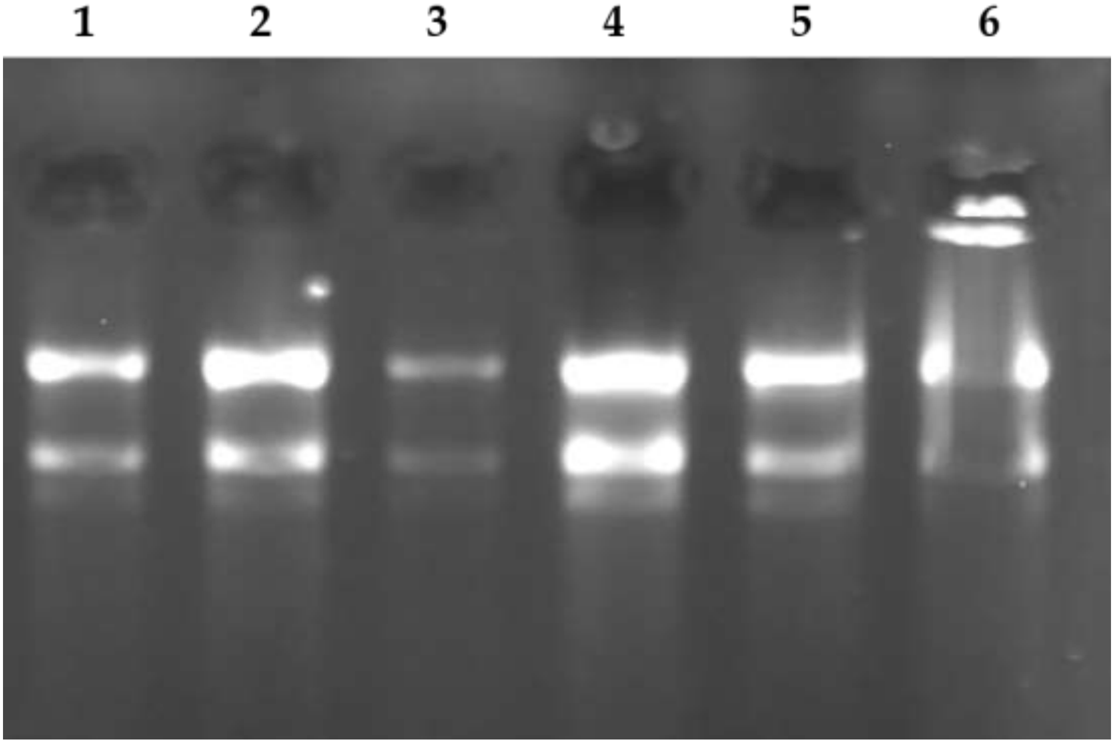

Figure S10 Extraction of RNA from soybean *GmSNF1* gene-silenced plants under salt and salt-alkali stress. 1, 3, and 5 represent for control plants under no treatment, salt treatment, and salt-alkali treatment, respectively; 2, 4, and 6 represent *GmSNF1* gene-silenced plants under no treatment, salt treatment, and salt-alkali treatment, respectively (The degraded RNA of some samples, such as 3 and 6, had been re-extracted for the experiment.).
